# Bee venom acupuncture therapy ameliorates ulcerative colitis by reducing inflammatory response, oxidative stress, and suppressing the NF-κB/HIF-1α pathway

**DOI:** 10.1515/biol-2025-1326

**Published:** 2026-05-05

**Authors:** Jing Liang, Xu Ma, Yuqing Ren, Mo Chen, Huan Ma, Jingbo Guo

**Affiliations:** Department of Traditional Chinese Medicine, Bethune International Peace Hospital of PLA, Shijiazhuang, Hebei, China

**Keywords:** ulcerative colitis, bee venom, acupuncture, inflammatory response, oxidative stress, NF-κB/HIF-1α pathway

## Abstract

Ulcerative colitis (UC) is a chronic inflammatory bowel disease that poses a significant challenge to public health due to its relapsing nature and complex pathogenesis. This study investigated the protective effects of bee venom acupuncture on UC and explored the underlying mechanisms using a rat model of UC. The findings demonstrated that bee venom acupuncture significantly restored body weight, reduced disease activity index (DAI) scores, and ameliorated colonic tissue injury. Moreover, bee venom therapy decreased serum levels of tumor necrosis factor-α (TNF-α), interleukin-1β (IL-1β), interleukin-6 (IL-6), and interleukin-8 (IL-8), and downregulated their protein expression in colon tissue. Furthermore, assessment of oxidative stress biomarkers revealed that bee venom treatment significantly reduced reactive oxygen species (ROS) and malondialdehyde (MDA) levels while elevating superoxide dismutase (SOD), glutathione (GSH), and catalase (CAT) levels in the colon. Western blot and immunohistochemistry analyses indicated that bee venom treatment inhibited NF-κB/HIF-1α pathway. In conclusion, bee venom acupuncture ameliorates UC by reducing inflammatory responses, alleviating oxidative stress, and suppressing NF-κB/HIF-1α pathway, suggesting its potential as a therapeutic strategy for UC management.

## 1 Introduction

Ulcerative colitis (UC) is characterized by continuous, diffuse inflammation and ulceration of the colonic mucosa. Clinically, it manifests as abdominal pain, diarrhea, mucous and bloody stools, and weight loss [1]. Current clinical management of UC primarily relies on glucocorticoids and immunosuppressants. However, these therapies are limited by poor targeting, significant adverse effects, and unfavorable prognoses [2]. Consequently, further elucidation of UC pathogenesis and the development of novel treatment strategies are essential for enhancing patients’ quality of life.

Bee venom is a complex biological mixture derived from honeybees, comprising various proteins, peptides, and amino acids. These components exhibit various pharmacological characteristics, including anti-inflammatory, antioxidant, analgesic, antibacterial, and antiviral properties, demonstrating significant therapeutic potential for various inflammatory diseases [3]. For instance, melittin, a principal component of bee venom, was shown to ameliorate cisplatin-induced acute kidney injury in mice by modulating M2 macrophage activation [4]. Similarly, apamin alleviates gouty arthritis by inhibiting pro-inflammatory cytokine production and inflammasome formation [5]. Furthermore, bee venom exhibits notable antioxidant activity, previous study indicated that it reduced levels of nitric oxide (NO) and malondialdehyde (MDA) while increasing total antioxidant capacity (TAC), glutathione (GSH), and paraoxonase-1 (Pon-1), thereby mitigating oxidative stress in Parkinson’s disease [6]. Another study demonstrated that bee venom protected neural cells against amyloid-beta-induced oxidative stress by modulating the Nrf2/HO-1 pathway [7]. However, the specific role of bee venom acupuncture in UC and its underlying molecular mechanisms remain inadequately understood.

Nuclear factor kappa B (NF-κB) is a critical nuclear transcription factor essential for maintaining intestinal immune homeostasis [8]. Aberrant activation of NF-κB pathway is closely associated with the pathogenesis of UC, triggering the release of pro-inflammatory cytokines and initiating a cascade of inflammatory responses and mucosal damage [9]. Hypoxia-inducible factor-1 (HIF-1) is a transcriptionally active heterodimeric nuclear protein composed of HIF-1α and HIF-1β. The HIF-1α subunit, is crucial for oxygen regulation and plays a pivotal role in intestinal metabolism and barrier maintenance [10]. Research has demonstrated that HIF-1α is regulated by several transcription factors, with NF-κB serving a central role, p-NF-κB dimers translocate to the nucleus to enhance HIF-1α transcription [11]. Consequently, inhibiting NF-κB/HIF-1α pathway is essential for alleviating UC. For example, berberine was shown to reduce colonic inflammatory responses and improved UC in mice by suppressing TLR4/NF-κB/HIF-1α pathway [12]. Similarly, Jian-Pi Qing-Chang decoction alleviated UC by regulating NF-κB/HIF-1α pathway, thereby ameliorating intestinal mucosal inflammation [13].

The present study aimed to investigate the therapeutic effects of bee venom acupuncture on UC and to elucidate its underlying mechanisms. Our findings indicate that bee venom acupuncture alleviates UC in rats by inhibiting inflammatory responses, attenuating oxidative stress, and suppressing NF-κB/HIF-1α pathway. This research presents an innovative strategy for UC treatment and offers new insights into the clinical application of bee venom.

## 2 Materials and methods

### 2.1 Reagents

Bee venom, with a purity of ≥95 % and a melittin content of 60–75 %, was purchased from North China Pharmaceutical Co., Ltd.; TNBS was obtained from Sigma-Aldrich (USA), and hematoxylin and eosin were sourced from Solarbio (China). ELISA kits for tumor necrosis factor-α (TNF-α), interleukin-1β (IL-1β), interleukin-6 (IL-6), and interleukin-8 (IL-8) were procured from Jiangsu Jingmei (China). Assay kits for reactive oxygen species (ROS), MDA, superoxide dismutase (SOD), GSH, and catalase (CAT) were acquired from Beyotime (China). Primary antibodies against TNF-α, IL-1β, IL-6, IL-8, HIF-1α, and β-actin, as well as the secondary antibody, were purchased from Abcam (UK). The p-NF-κB antibody was obtained from MCE (USA), and the NF-κB antibody was sourced from CST (USA).

### 2.2 Animals

Sixty-five Wistar rats (200–220 g, 33 males and 32 females), were procured from Beijing SPF Biotechnology Co., Ltd. The rats were housed in a controlled laboratory environment (temperature: 23 ± 3 °C; relative humidity: 50 ± 5 %; 12 h light/dark cycle). Both food and water were continuously accessible to them.

**Ethical approval:** The research related to animal use has been complied with all the relevant national regulations and institutional policies for the care and use of animals, and has been approved by the Medical Ethics Committee of the 980th Hospital of the PLA Joint Logistic Support Force (2022-KY-33).

### 2.3 UC model establishment and experimental grouping

Following a 3-day acclimatization period, 15 rats were randomly selected as the normal group, while the remaining animals were used to establish UC model via the TNBS/ethanol enema method. Following a 24 h fasting period, the rats were anesthetized via intraperitoneal injection of pentobarbital. While anesthetized, the animals were placed in a head-down prone position, and a polyethylene catheter with a 2 mm diameter was placed 8 cm into the rectum. A TNBS/ethanol solution (0.25 mL, 100 mg/kg) was slowly infused, this enema procedure was repeated for three consecutive days. On the fourth day, five rats were randomly sacrificed to collect colon tissues for histological validation of successful UC induction. On day 5, the remaining 45 successfully modeled rats were randomly divided into three groups to initiate treatment: the model group: no treatment, the control group: daily injection of sterile water (0.1 mL/site) at bilateral Tianshu (ST25) and Zusanli (ST36) acupoints, the bee venom group: daily injection of bee venom (0.05 mg/kg, 0.1 mL/site) at the same acupoints. Seven days post-treatment, all rats were euthanized via an intraperitoneal overdose of pentobarbital, and colon tissues were harvested for subsequent analysis.

### 2.4 Hematoxylin and eosin (HE) staining

Colonic tissues preserved in formaldehyde were subjected to standard paraffin embedding, and then sliced into 5 μm thick sections, stained with hematoxylin and eosin, and images were taken at 200× magnification [14].

### 2.5 Detection of TNF-α, IL-1β, IL-6, and IL-8 levels in serum

Serum levels of TNF-α, IL-1β, IL-6, and IL-8 were quantified using ELISA kits according to the manufacturer’s instructions. The absorbance was measured at a wavelength of 450 nm using a microplate reader (Thermo Fisher Scientific, USA) [15].

### 2.6 Detection of ROS, MDA, SOD, GSH, and CAT levels in colonic tissue

Fresh colon tissues were precisely weighed and homogenized in ice-cold PBS at a ratio of 1:9 (w/v) to prepare 10 % tissue homogenates using a tissue grinder (Shanghai Jingxin, China) maintained at 4 °C. Following centrifugation, the supernatant was collected. The levels of ROS, MDA, SOD, GSH, and CAT in the colon tissues were quantified using commercial assay kits in strict accordance with the manufacturer’s instructions.

### 2.7 Western blot (WB)

Proteins were isolated with lysis buffer and their concentration was assessed through the BCA assay. Equivalent amounts of protein were separated using SDS-PAGE and transferred onto PVDF membranes. The membranes were blocked and subsequently incubated with primary antibody overnight at 4 °C. Following three washes with TBST, the membranes were incubated with the secondary antibody at room temperature for 1 h. After washing, the membranes were developed using ECL detection solution. Images were captured using a chemiluminescence imaging system, and grayscale analysis was performed with Image J software [16]. The antibody dilution ratios were as follows: TNF-α (1:1,000), IL-1β (1:2,000), IL-6 (1:2,000), IL-8 (1:2,000), p-NF-κB (1:1,000), NF-κB (1:2,000), HIF-1α (1:1,000), β-actin (1:5,000), and secondary antibody (1:3,000).

### 2.8 Immunohistochemistry (IHC)

Paraffin-embedded sections were deparaffinized and subjected to antigen retrieval. Following rinsing with PBS, the sections were blocked with serum for 30 min. After being incubated overnight at 4 °C with the primary antibody, the sections were rinsed with PBS and then the secondary antibody was applied. Subsequently, DAB chromogenic solution was added for a 5-min incubation. The sections were counterstained with hematoxylin, dehydrated, and cover slipped. Images were captured using a microscope (magnification, 200×) [17]. The primary antibody dilution ratios for IHC were as follows: TNF-α (1:500), IL-1β (1:500), IL-6 (1:300), IL-8 (1:200), p-NF-κB (1:200), NF-κB (1:400), HIF-1α (1:400), and β-actin (1:500). The secondary antibody was used at a dilution of 1:200.

### 2.9 Statistical analysis

GraphPad Prism 9.0 was used to analyze all data, and the results are shown as mean values with standard deviation (SD). An independent samples *t*-test was used for comparing two groups, and one-way ANOVA was applied for comparisons among multiple groups. Statistical significance was set at *P* < 0.05.

## 3 Results

### 3.1 Bee venom acupuncture improves UC in rats

**Figure 1:**
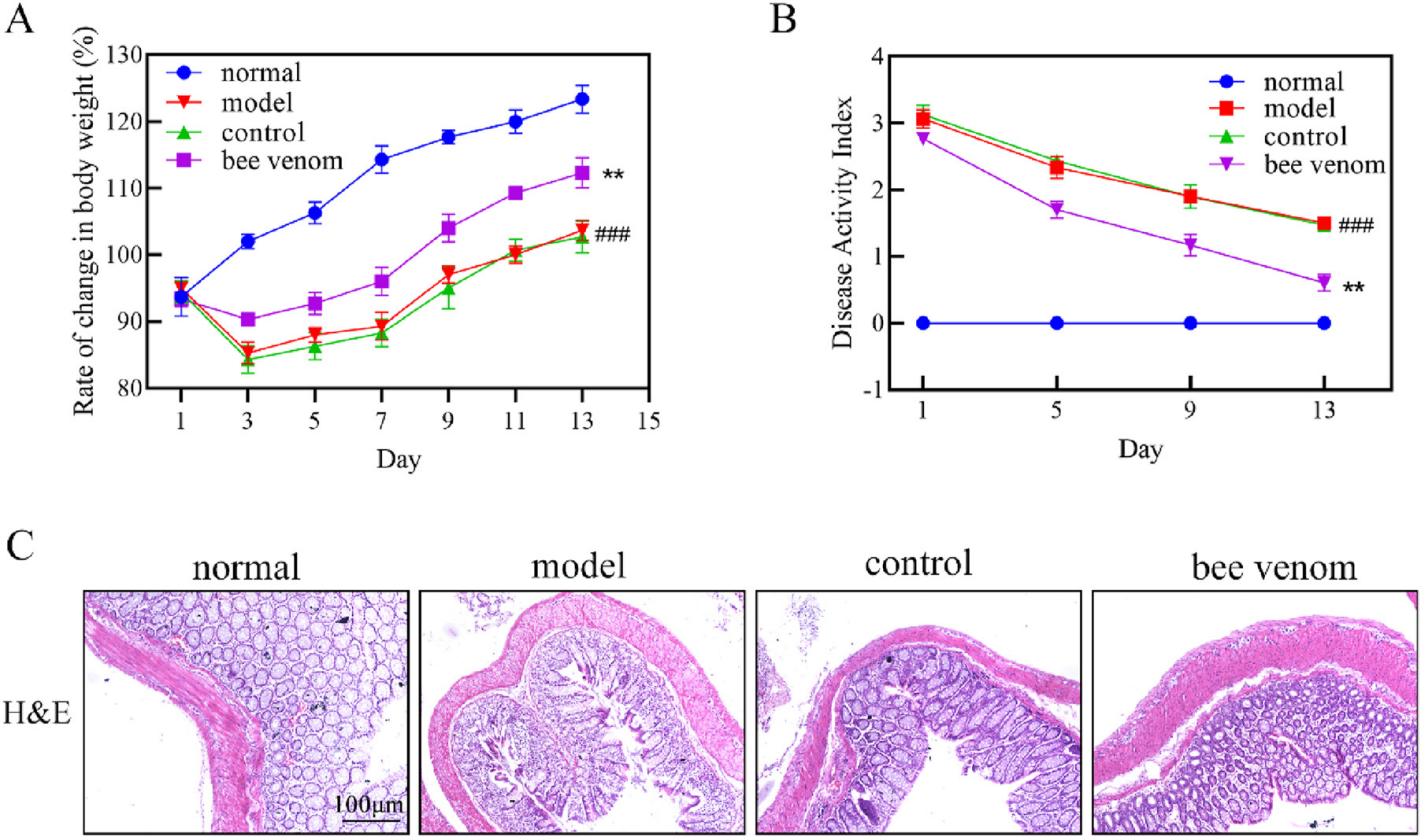
Bee venom acupuncture alleviates UC in rats. (A) Rate of change in body weight for each group of rats. (B) DAI for each group of rats. (C) HE staining of colonic tissue from each group of rats (200×). Data are presented as the mean ± SD, ^###^ *P* < 0.001, compared with normal group, ^**^ *P* < 0.01 compared with model group.

We established a UC rat model using the TNBS/ethanol enema method to assess the impact of bee venom acupuncture on UC. The findings showed that bee venom therapy notably enhanced body weight change rates and reduced the Disease Activity Index (DAI) **(** Figure 1A and B). HE staining revealed that the colonic mucosa of rats in the normal group exhibited a smooth surface, intact structure, and orderly arrangement of epithelial cells, with no inflammatory cell infiltration detected. Conversely, the model group displayed severe colonic mucosal damage, disorganized glandular arrangement, a reduction in goblet cells, and significant inflammatory cells infiltration. The control group also showed colonic mucosal damage, destruction of intestinal glands, and widespread inflammatory infiltration. However, the bee venom group displayed a well-preserved intestinal mucosa with intact glands and minimal inflammatory cell presence (Figure 1C). These findings suggest that bee venom acupuncture could ameliorate colonic tissue damage in UC rats.

### 3.2 Bee venom acupuncture inhibits inflammatory response in UC rats

**Figure 2:**
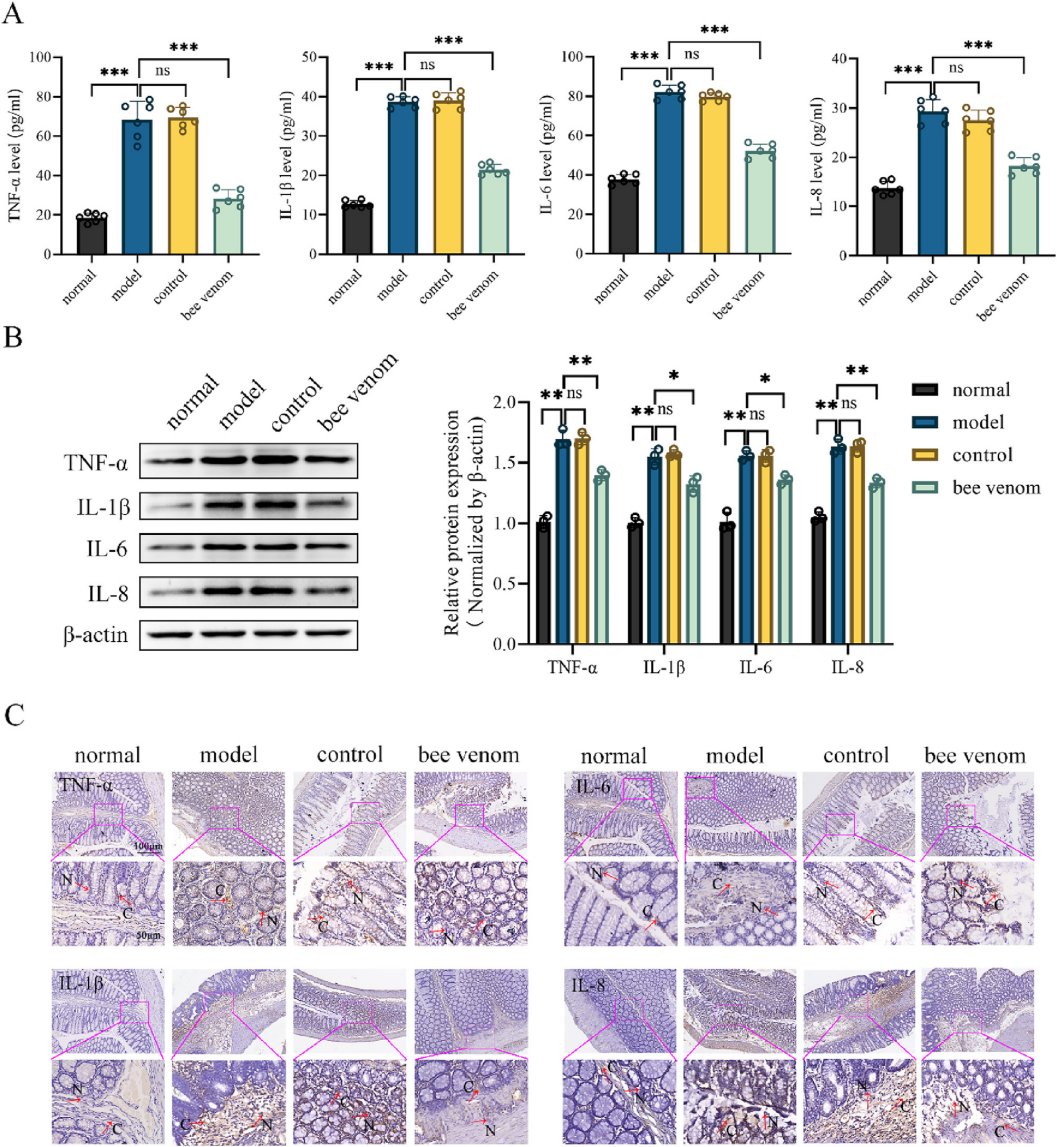
Bee venom acupuncture’s effects on inflammatory response in UC rats. (A) TNF-α, IL-1β, IL-6, and IL-8 levels in the serum of each group of rats. (B) Protein expression of TNF-α, IL-1β, IL-6, and IL-8 in colon tissues of each group of rats. (C) IHC images of TNF-α, IL-1β, IL-6, and IL-8 in colon tissues of each group of rats (200×). Data are presented as the mean ± SD, ^*^ *P* < 0.05, ^**^ *P* < 0.01, ^***^ *P* < 0.001.

Given the close association between UC and inflammatory responses, we utilized ELISA to evaluate pro-inflammatory factor levels in serum. The results indicated a significant elevation in TNF-α, IL-1β, IL-6, and IL-8 in the model group, while bee venom acupuncture significantly reduced the levels of these cytokines in UC rats (Figure 2A). Furthermore, WB results revealed that the protein expression levels of TNF-α, IL-1β, IL-6, and IL-8 in the colonic tissues were notably increased in the model group **(** Figure 2B and C). Specifically, IHC analysis demonstrated intense positive staining for these cytokines in the colon tissues of the model group. In contrast, bee venom acupuncture markedly decreased the expression of these proteins, as evidenced by significantly weaker staining intensity. These findings indicate that bee venom acupuncture effectively mitigates the inflammatory response in rats with UC.

### 3.3 Bee venom acupuncture reduces oxidative stress in UC rats

**Figure 3:**
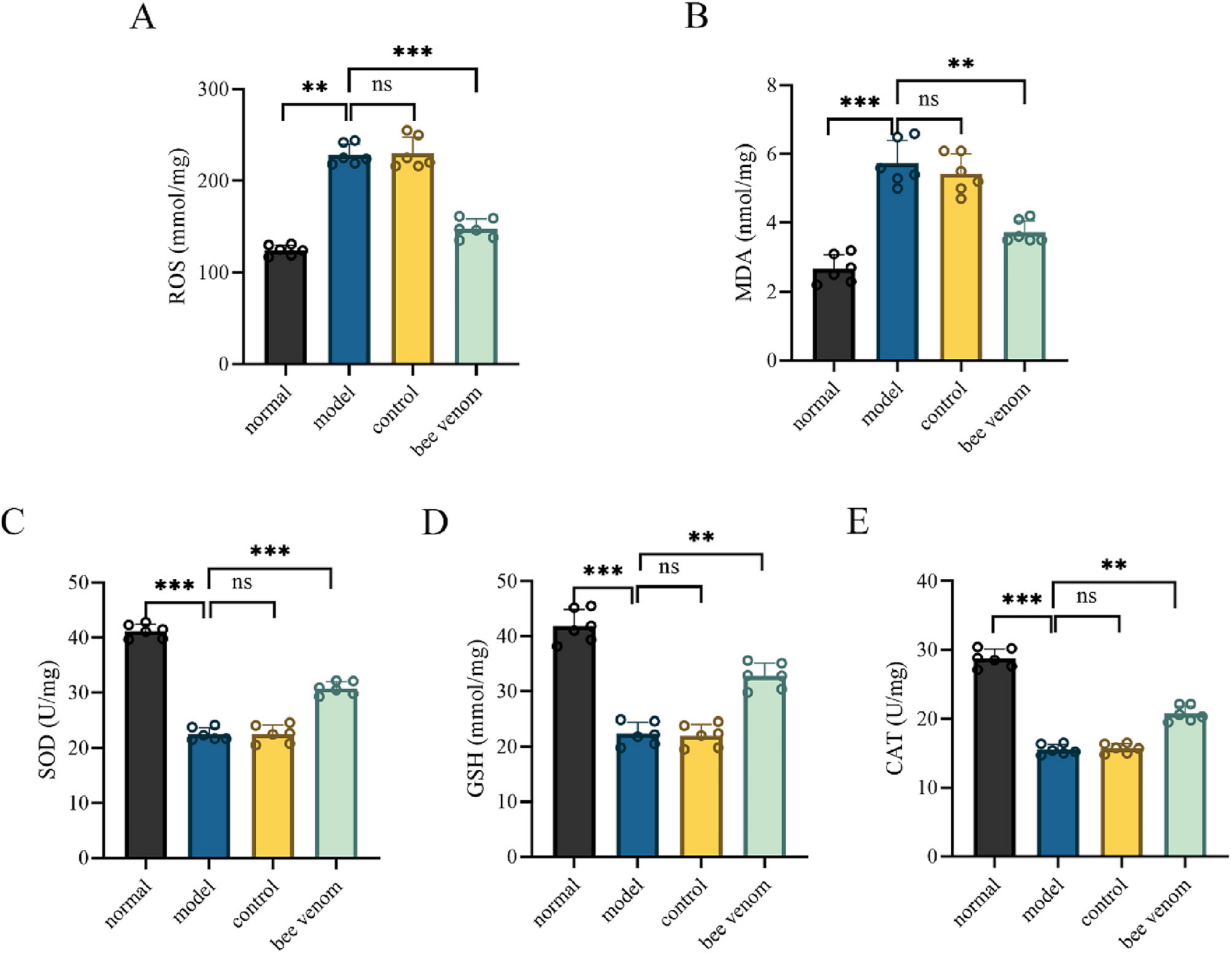
The role of bee venom acupuncture in managing oxidative stress in UC rats. (A–E) ROS, MDA, SOD, GSH, and CAT levels in colon of each group of rats. Data are presented as mean ± SD, ^*^ *P* < 0.05, ^**^ *P* < 0.01, ^***^ *P* < 0.001.

To investigate the impact of bee venom acupuncture on oxidative stress in UC rats, colonic levels of ROS, MDA, SOD, GSH, and CAT were measured. The results demonstrated that ROS and MDA levels were significantly elevated in the colon tissues of the model group, while the levels of SOD, GSH, and CAT were significantly reduced (Figure 3A–E). Following bee venom treatment, the colonic levels of ROS and MDA were significantly decreased, whereas the levels of SOD, GSH, and CAT were significantly increased (Figure 3A–E). These findings suggest that bee venom acupuncture effectively inhibits oxidative stress in the colons of UC rats.

### 3.4 Bee venom acupuncture improves UC in rats by suppressing NF-κB/HIF-1α pathway

**Figure 4:**
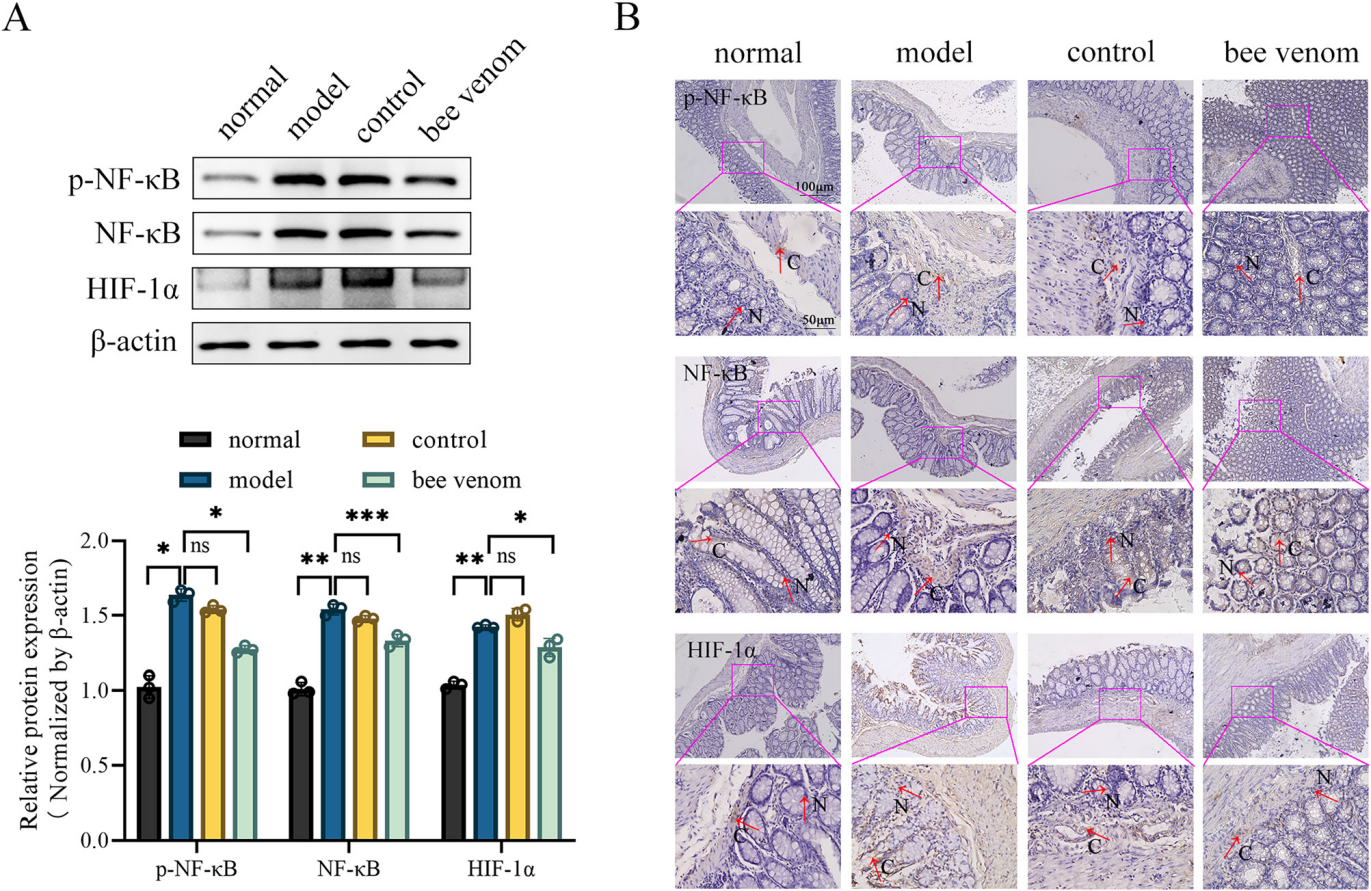
Impact of bee venom acupuncture on NF-κB/HIF-1α pathway in UC rats. (A) Protein levels of p-NF-κB, NF-κB, and HIF-1α in the colon tissues of each group of rats. (B) IHC images of p-NF-κB, NF-κB, and HIF-1α in the colon tissues of each group of rats (200×). Data are presented as the mean ± SD, ^*^ *P* < 0.05, ^**^ *P* < 0.01, ^***^ *P* < 0.001.

Given the importance of the NF-κB/HIF-1α pathway in UC pathogenesis, we evaluated the impact of bee venom treatment on the expression of related proteins via WB. The findings showed that p-NF-κB, NF-κB, and HIF-1α protein expression in the colon tissues of the model group was significantly elevated **(** Figure 4A). Conversely, p-NF-κB, NF-κB, and HIF-1α protein expression was significantly decreased in the bee venom group (Figure 4A). Consistent with the WB findings, IHC staining demonstrated strong immunoreactivity for p-NF-κB, NF-κB, and HIF-1α in the colon tissues of the model group. Following bee venom treatment, the immunoreactivity of these proteins was significantly reduced, which was characterized by lighter staining and a decrease in the number of positive cells (Figure 4B). These results suggest that bee venom treatment effectively inhibits the stimulation of NF-κB/HIF-1α pathway induced by UC.

## 4 Discussion

UC is defined by chronic, nonspecific inflammatory lesions localized to the rectal and colonic mucosa and submucosa [18]. Patients with UC frequently experience recurrent and persistent episodes of diarrhea, mucous and bloody stools, and abdominal pain. Recently, the global incidence of UC has steadily increased, establishing it as a major global public health concern. Consequently, elucidating the pathogenesis of UC and developing effective therapeutic strategies are crucial for improving patients’ quality of life. Bee venom, secreted by the venom glands of bees, comprises various bioactive components with diverse pharmacological properties, demonstrating efficacy in treating inflammatory diseases such as arthritis, dermatitis, and periodontitis. The most common apitherapy approach involves the injection of low concentrations of bee venom at acupoints. Research has indicated that the injection of bee venom at the Zusanli (ST36) acupoint can decrease the accumulation of alpha-synuclein (α-syn) in the brain by inhibiting neuroinflammation and oxidative stress [19]. Nevertheless, the precise role and molecular mechanisms of bee venom acupuncture in UC remain inadequately understood. Therefore, we established a UC rat model to investigate the therapeutic effects and underlying mechanisms of bee venom acupuncture. Our findings revealed that bee venom therapy significantly improved body weight changes and reduced the DAI in UC rats. Furthermore, HE staining illustrated that bee venom treatment mitigated colonic mucosal damage, alleviating histopathological features such as disorganized gland arrangement and inflammatory cell infiltration.

An imbalance between pro-inflammatory and anti-inflammatory signals is a hallmark of intestinal mucosal inflammation in UC. Cytokines are crucial in UC development and represent potential therapeutic targets [20]. TNF-α, produced by various immune cell subsets, stimulates the release of cytokines, chemokines, and ROS during chronic intestinal inflammation, anti-TNF-α therapies are widely employed in UC treatment [21]. Notably, the concentration of TNF-α positively correlates with the number of inflammatory cells and the degree of vascularization in pathological tissues [22]. Furthermore, TNF-α exerts double-sided effect, while lower concentrations promote cell proliferation, higher concentrations induce cell death [23]. In the context of UC, excessive TNF-α production drives sustained inflammatory cell infiltration and epithelial damage. In our study, bee venom treatment significantly reduced TNF-α levels in both serum and colon tissues, correlating with attenuated inflammatory cell infiltration and ameliorated colonic mucosal damage. IL-1β, a pivotal pro-inflammatory cytokine, increases markedly during pathological processes and triggers the release of IL-6, IL-8, and other inflammatory factors [24]. IL-6 and IL-8 function as chemokines that are critical for neutrophil recruitment to inflammatory sites. Infiltrating neutrophils release inducible nitric oxide synthase and matrix metalloproteinases, thereby exacerbating mucosal inflammation and epithelial damage [25]. In the present study, bee venom treatment significantly decreased serum levels of IL-1β, IL-6, and IL-8 in UC rats. These findings were corroborated by WB and IHC, which demonstrated reduced protein expression of these cytokines in colon tissues. Collectively, these results indicate that bee venom treatment alleviates UC-associated inflammation by suppressing the production of key pro-inflammatory cytokines.

Oxidative stress is a primary mechanism driving the onset and progression of UC. Inflammatory responses within the colon stimulate excessive production of ROS, which damages the body’s oxidative defense system, leading to oxidative stress-related tissue injury. This disruption of the intestinal mucosa facilitates pathogen invasion and further exacerbates inflammatory responses [26]. Clinical studies have demonstrated that UC patients exhibit significantly elevated levels of oxidative stress biomarkers in both serum and colonic mucosal tissues compared to healthy individuals [27], 28]. Additionally, research has indicated that lithospermic acid could alleviate UC by increasing the production of SOD, GSH-PX, and CAT, while simultaneously reducing ROS and MDA levels [29]. Therefore, maintaining systemic redox balance is crucial for inhibiting UC progression. In the present study, bee venom treatment significantly reduced colonic ROS and MDA levels, while concurrently elevating SOD, GSH, and CAT levels, thereby mitigating UC-induced oxidative stress.

NF-κB serves as a critical transcription factor that regulates various inflammatory responses. Research indicates that preventing NF-κB activation may reduce the production of inflammatory factors, thereby alleviating UC symptoms [30]. Similarly, HIF-1α acts as an essential transcription factor for preserving the intestinal barrier integrity and modulating immune responses [31]. The expression of HIF-1α is notably elevated in the colons of UC patients, and this upregulation is modulated by NF-κB [32]. For instance, one study demonstrated that mango polyphenolics alleviate UC by inhibiting the protein expression of NF-κB, p-NF-κB, and HIF-1α [33]. Furthermore, Fuzi-Baijiangcao herbal medicine has been shown to improve intestinal damage in UC mice through suppressing NF-κB/HIF-1α pathway [34]. In this study, we assessed the expression of proteins related to NF-κB/HIF-1α pathway in colon tissues using WB and IHC. The results indicated that bee venom treatment significantly reduced the protein expression of p-NF-κB, NF-κB, and HIF-1α in the colon. Thus, bee venom acupuncture therapy may alleviate UC in rats through suppressing NF-κB/HIF-1α pathway.

In summary, our study demonstrates that bee venom acupuncture therapy could prevent UC progression. This effect is achieved through the suppression of inflammatory responses and oxidative stress, alongside the regulation of NF-κB/HIF-1α pathway. These findings offer novel theoretical support for the potential clinical application of bee venom acupuncture in the treatment of UC.
